# The Interface amongst Conserved and Specialized Pathways in Non-Paclitaxel and Paclitaxel Accumulating *Taxus* Cultures

**DOI:** 10.3390/metabo11100688

**Published:** 2021-10-07

**Authors:** Michelle C. McKee, Sarah A. Wilson, Susan C. Roberts

**Affiliations:** 1 Biology & Biotechnology, Worcester Polytechnic Institute, Worcester, MA 01609, USA; 2 Chemical Engineering, University of Massachusetts Amherst, Amherst, MA 01003, USA; 3 Chemical Engineering, Worcester Polytechnic Institute, Worcester, MA 01609, USA

**Keywords:** plant cell culture, taxane biosynthesis, metabolic flux

## Abstract

Plant cell cultures derived from *Taxus* are used to produce valuable metabolites like paclitaxel, a chemotherapeutic drug. Methyl jasmonate elicitation enhances paclitaxel accumulation, but also inhibits culture growth and increases phenylpropanoid biosynthesis, two side effects that detract from taxane accumulation. To understand the connection between all of these processes, a systems approach is applied to investigate cell-wide metabolism in *Taxus*. Non-paclitaxel and paclitaxel accumulating cultures were elicited over single and multi-generational periods, and subsequent changes in conserved and specialized metabolism were quantified. Methyl jasmonate typically resulted in decreased growth and increased metabolite content in paclitaxel accumulating cultures. Conversely, elicitation typically resulted in either no change or decrease in accumulation of metabolites in the non-paclitaxel accumulating cultures. In both sets of cultures, variability was seen in the response to methyl jasmonate across generations of cell growth. Consolidation of these data determined that paclitaxel accumulation and basal levels of phenolic and flavonoid compounds are indirectly correlated with aggregate size. These approaches assess alternative metabolic pathways that are linked to paclitaxel biosynthesis and provide a comprehensive strategy to both understand the relationship between conserved and specialized metabolism in plants and in the design of strategies to increase natural product yields in plant cell culture.

## 1. Introduction

Plant specialized (secondary) metabolism provides an incredible assortment of natural products that can be used as pharmaceuticals, nutraceuticals, flavors, fragrances, and pesticides. This chemical diversity is the result of millions of years of evolution duplicating, extending, and modifying biosynthetic pathways containing unique combinations of enzymes and precursors [1]. These compounds confer environmental-specific benefits that conserved (primary) metabolic pathways, or those for growth and development, cannot provide, such as pathogen protection, herbivore deterrence, and pollinator attraction. The conserved metabolic pathways are well understood and consistent across plant species; they often generate the precursors that feed into specialized metabolite biosynthetic pathways. Specialized metabolite biosynthesis remains enigmatic due to the vastness of the plant kingdom. To truly understand species-specific metabolism, each would require genomic, transcriptomic, and metabolomic studies, and often, these vary across different plant tissues and time, making that an arduous and expensive undertaking. However, there are other tangible approaches to understand how specialized metabolites are derived from conserved metabolism and how to influence flux between the two to increase metabolite accumulation.

**Figure 1:**
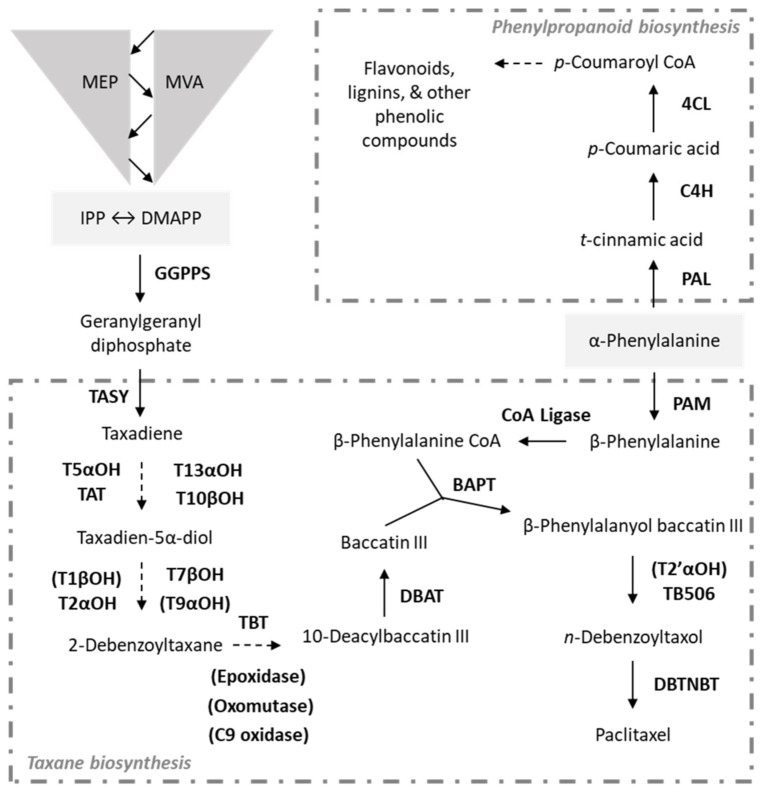
Metabolic map for key specialized metabolic pathways in *Taxus* cell culture. Paclitaxel biosynthesis feeds from the interchanging plastidic MEP and cytosolic MVA pathways. Hypothesized and unidentified enzymes are denoted with parentheses. Enzyme abbreviations: geranylgeranyl pyrophosphate synthase (GGPPS), taxadiene synthase (TASY), taxane 5α-hydroxylase (T5αOH), taxadien-5α-ol-O-acetyl-transferase (TAT), taxane 13α-hydroxylase (T13αOH), taxane 10β-hydroxylase (T10βOH), taxane 1β-hydroxylase (T1βOH), taxane 2α-hydroxylase (T2αOH), taxane 7β-hydroxylase (T7βOH), taxane 9α-hydroxylase (T9αOH), taxane 2α-O-benzoyl transferase (TBT), 10-deacetyl-baccatin III-10-O-acetyltransferase (DBAT), phenylalanine aminomutase (PAM), 13-O-(3-amino-3-phenylpropanoyl) transferase (BAPT), putative hydroxylase (TB506), 3′-N-debenzoyl-2′-deoxytaxol-N-benzoyltransferase (DBTNBT), phenylalanine lyase (PAL), cinnamate 4-hydroxylase (C4H), 4CL 4-coumarate CoA ligase (4CL).

Plant cell culture eliminates tissue-specific metabolisms and is used to study metabolism under conditions that can be tightly controlled, replicated, and manipulated [2]. In addition to a research tool, plant cell cultures can be used as production platforms for valuable natural products that cannot be sustainably supplied by its native species or via chemical synthesis [3]. *Taxus*, the yew tree genera, has been studied as a culture with the goal of increasing production of its specialized metabolite, paclitaxel. Paclitaxel (Taxol^®^) is an important microtubule stabilizing drug used for the treatment of breast, ovarian, and lung cancer, as well as Kaposi’s sarcoma [4]. Paclitaxel, a triterpenoid, is a product of the taxane biosynthetic pathway starting with isopentyl pyrophosphate (IPP) and dimethylallyl pyrophosphate (DMPP) from the plastidic MEP and cytosolic MVA pathways, which are conserved and interchanging [5] (Figure 1). Within the hypothesized paclitaxel biosynthetic pathway, 14 out of the putative 19 enzymatic steps have been identified [6]. The phenylpropanoid biosynthetic pathway competes with that of taxanes as they share the phenylalanine precursor pool (Figure 1). Phenylpropanoid products in *Taxus* include phenolic acids, flavonoids, and lignin, all of which are considered specialized metabolites [7,8,9]. As paclitaxel is the yield goal of *Taxus* cultures commercially, researchers have taken several approaches to understand the metabolic pathways of all compounds and to manipulate flux towards paclitaxel synthesis.

There are many controllable factors that have been optimized for specialized metabolism in *Taxus* culture. Past research has investigated different *Taxus* species and media composition and their influence on culture growth and paclitaxel accumulation [10,11]. The size of aggregates in a culture has shown to influence paclitaxel production, where smaller aggregates accumulate more paclitaxel [12,13]. Another simple method to shift metabolic flux from conserved to specialized metabolism is elicitation [14,15]. Even with the many elicitors explored in *Taxus* culture, methyl jasmonate remains the most useful and common; it consistently induces an increase in paclitaxel accumulation along with other taxanes through upregulation of genes related to taxane biosynthesis [15,16,17]. However, methyl jasmonate elicitation does not affect taxane biosynthesis exclusively as it acts broadly on specialized metabolism, meaning it also increases phenylpropanoid biosynthesis as evident through transcriptomic and metabolomic analysis of *Taxus* [7,16,18,19]. As specialized metabolism increases as a result of elicitation, overall culture growth is typically repressed and cell cycle progression is stalled [20,21]. This decrease is expected as metabolic flux is redirected and pulls from conserved metabolism. To further increase paclitaxel accumulation beyond the boost methyl jasmonate provides, a systemic approach to comprehensively study specialized metabolism is needed instead of hyper-focusing on the desired metabolite and directly related pathway. The relationship between taxanes and other classes of specialized products including phenylpropanoids needs to be elucidated to isolate the effects of elicitation. In addition to the shared phenylalanine precursor pool, the pathways are likely tied through gene expression regulation or overlapping feedback signals, which are observed in other plant metabolic pathways [22]. However, uncovering the differences amongst the pathways will provide new opportunities to manipulate specialized metabolic flux towards paclitaxel in *Taxus* culture.

This paper uses *Taxus* non-paclitaxel accumulating cell lines along with paclitaxel accumulating cell lines to understand the link between conserved and specialized metabolism, specifically the taxane and phenylpropanoid biosynthetic pathways, in *Taxus* culture. Non-paclitaxel accumulating cell lines are *Taxus* cultures that do not accumulate UPLC-detectable levels of paclitaxel even after elicitation, and elicitation does not inhibit their culture growth [23]. It is likely that epigenetic changes within the cultures inhibit the effects of methyl jasmonate on paclitaxel production [24,25]. Even though these cultures do not produce detectable levels of paclitaxel, they are able to synthesize phenylpropanoid compounds, and they were used to compare paclitaxel and non-paclitaxel accumulating cultures over short-term and generational time periods, a novel use of non-paclitaxel accumulating cultures. Through quantifying growth, paclitaxel accumulation, and phenylpropanoid accumulation parameters, this systems approach provides insight into the taxane and phenylpropanoid biosynthetic pathways in *Taxus*; results will likely be extendable to other specialized metabolic plant culture systems. This strategy provides new insights into the relationship between the taxane and phenylpropanoid metabolic pathways to identify new targets to increase paclitaxel accumulation in *Taxus* cell culture. A systems approach to understand specialized metabolism in plant culture systems has the potential to more broadly impact a variety of plant systems in contrast to pathway-specific analyses, which only benefit the specific system of study.

## 2. Results and Discussion

### 2.1. Methyl Jasmonate Treatment of Non-Paclitaxel and Paclitaxel Accumulating Cultures

Cell lines with different metabolic capacities, likely due to epigenetic variance, were used to comprehensively investigate *Taxus* metabolism. Non-paclitaxel (PO93X− and PO93XC−) and paclitaxel (P93AF+ and CO93D+) accumulating cultures were treated with methyl jasmonate and evaluated for differences in growth and metabolite accumulation. 14 days post elicitation, PO93X− and PO93XC− had not accumulated UPLC-detectable levels of paclitaxel as expected; whereas P93AF+ and CO93D+ had accumulated 0.05 mg/g dry weight and 0.15 mg/g dry weight paclitaxel, respectively.

**Figure 2:**
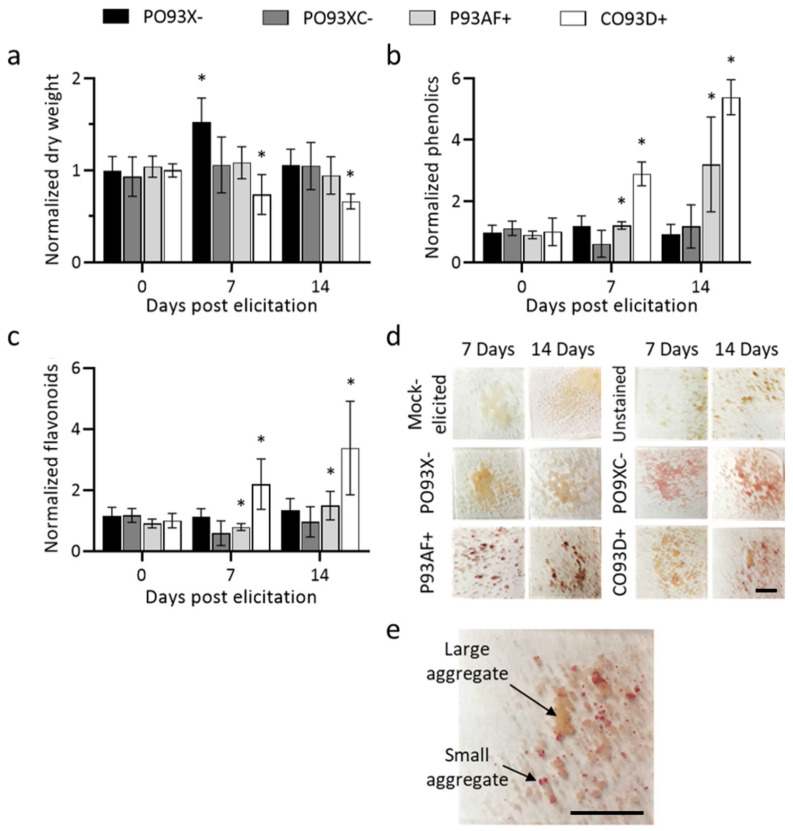
Effect of methyl jasmonate on paclitaxel and non-paclitaxel accumulating *Taxus* cultures. Dry weight (**a**), phenolic content (**b**), and flavonoid content (**c**) were quantified in PO93X−, PO93XC−, P93AF+, and CO93D+. These data were normalized to mock elicited controls. Lignin was stained using the phloroglucinol assay creating a red pigment in aggregates where lignin is present (scale bar is 5 mm) (**d**). Differential lignin accumulation from the CO93D+ culture in a magnified representative image (scale bar is 5 mm) (**e**). (*n* = 3 biological replicates, error bars = standard deviation) (Student’s *t*-test compared to 1, * *p* < 0.05).

Dry weight and metabolite content were quantified in elicited and unelicited cultures to measure culture growth and metabolic activity (Figure 2). The data are presented as the fold change of dry weight or metabolite accumulation due to elicitation. At the time of methyl jasmonate elicitation, there was no significant difference in biomass between the cultures (Figure 2a). PO93X− experienced dramatic culture growth in the first 7 days post elicitation; however, this effect dissipated 14 days after elicitation (Figure 2a). CO93D+ accumulated three times as much paclitaxel than P93AF+. This culture also showed a significant decrease in cell growth compared to the control culture after elicitation, indicating a negative impact of higher levels of paclitaxel accumulation on culture growth (Figure 2a).

Phenolic compounds, flavonoids, and lignin are end products of the phenylpropanoid pathway. Phenolic and flavonoid content remains unchanged between the non-paclitaxel accumulating lines after methyl jasmonate elicitation. In the two weeks post-elicitation, accumulation of these compounds increase up to 3-fold in P93AF+ and up to 5-fold in CO93D+ compared to mock elicited cultures (Figure 2b,c). This indicates that accumulation of the phenylpropanoid products, phenolics and flavonoids, closely mirror paclitaxel accumulation trends in the investigated cell lines.

Lignin, a structural phenylpropanoid, was qualitatively evaluated using an acidified phloroglucinol staining assay, and representative images are shown in Figure 2d. All cultures produced detectable lignin in response to methyl jasmonate elicitation, whereas the unelicited cultures did not. Methyl jasmonate has been shown to induce lignin production in plant cell culture [26], however this effect has not yet been reported in *Taxus* culture. Interestingly, the staining intensity was not uniform within all of the cultures; it varied among the aggregates. In particular, the COP3D+ culture shows that the smaller aggregates stained darker, indicating a higher concentration of lignin (Figure 2e). Past research has shown that smaller *Taxus* aggregates accumulate increased levels of paclitaxel [12,13]. This suggests that there could be an analogous effect with other specialized metabolic pathways such as phenylpropanoid biosynthesis; however, more information is needed to conclude this for lignin biosynthesis. In the future, lignin content could be measured using a quantitative assay in aggregates separated by size fractionation [27]. Lignin provides another upregulated phenylpropanoid product, in addition to phenolic and flavonoid compounds, in which cellular metabolic flux can be redirected via metabolic engineering to paclitaxel production.

These data indicate that methyl jasmonate elicitation shifts flux from conserved (as indicated by cell growth) to specialized metabolism (paclitaxel, phenolics, flavonoids and lignin) in the metabolically active, paclitaxel accumulating lines. Not only did CO93D+ accumulate the most paclitaxel, this culture’s biomass decreased most significantly over the 14 days post elicitation. This is consistent with previous research showing that unelicited *Taxus* cells accumulated more biomass, while culture growth slowed in those elicited with methyl jasmonate [20]. Additionally, the paclitaxel accumulating lines produced more phenolic and flavonoid compounds than the non-paclitaxel accumulating lines due to elicitation, which was expected due to the metabolic separation between paclitaxel and phenylpropanoid products (Figure 1). These data reaffirm that the methyl jasmonate response of these metabolic pathways are closely linked, but future experimentation should investigate the regulation of flux between conserved and specialized metabolism potentially as intracellular and extracellular signals such as hormones, proteins, and transcription factors. In other plant systems, exogenous methyl jasmonate has been shown to decrease growth and the transcription of growth-related genes, while increasing the levels of stress-related genes [28,29]. As more data is needed to understand the relationship between the two specialized metabolite pathways in *Taxus*, it is recommended to use both paclitaxel and non-paclitaxel accumulating cultures for these efforts.

### 2.2. The Effect of Methyl Jasmonate Treatment in Different Generations

To understand the variability of long-term culture elicitation, PO93XC− and P93AF+, were monitored over 9 generations for growth and metabolic activity. Each generation consisted of elicited cultures and unelicited cultures; the unelicited cultures were subcultured to initiate the subsequent generation. Table 1 details the paclitaxel content in P93AF+ over the odd-numbered generations 7 and 14 days post-methyl jasmonate elicitation. Paclitaxel accumulation remains consistent in this period with the exception of generation 7, which is significantly lower (one of the three biological replicates even had no detectible paclitaxel). This is not surprising as variability in paclitaxel accumulation over generations in *Taxus* culture has been observed before [13]. However, the simultaneous effects on other metabolic pathways remains unknown.

**Figure 3:**
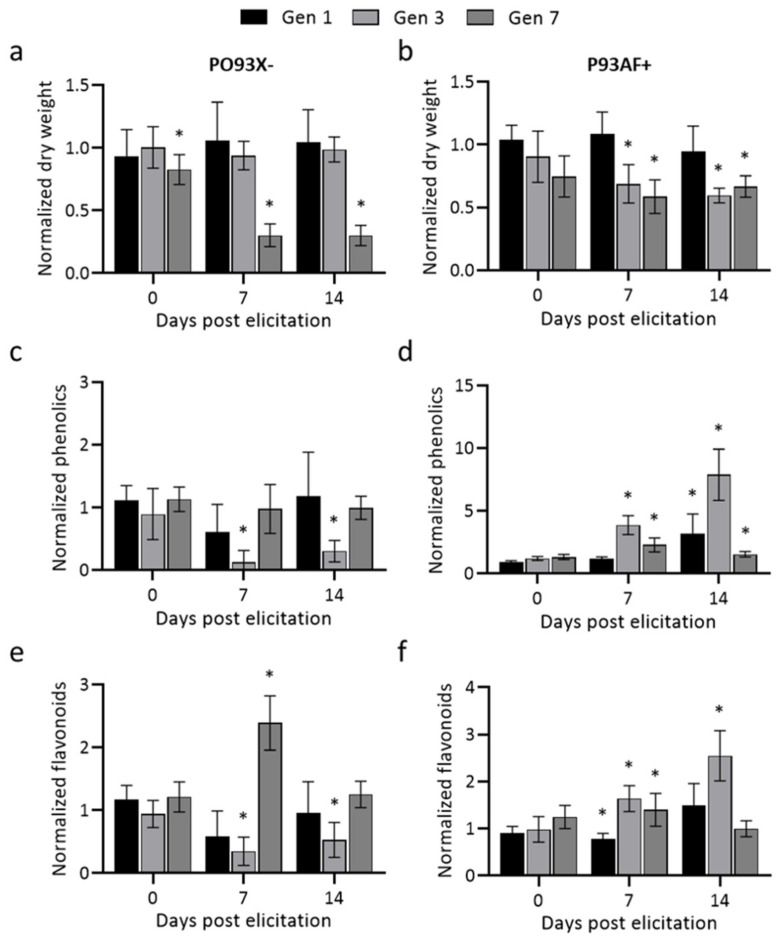
Long-term effect of methyl jasmonate on paclitaxel and non-paclitaxel accumulating *Taxus* cultures. Dry weight (**a**,**b**), phenolic content (**c**,**d**), and flavonoid content (**e**,**f**) were quantified in PO93XC− (**a**,**c**,**e**) and P93AF+ (**b**,**d**,**f**). These data were normalized to mock elicited controls. (*n* = 3 biological replicates, error bars = standard deviation) (Student’s *t*-test compared to 1, * *p* < 0.05).

Figure 3 shows the growth and specialized metabolite data for methyl jasmonate elicited PO93XC− and P93AF+ on generations 1, 3, and 7; data were normalized to the unelicited controls as in Figure 2. Methyl jasmonate did not affect culture growth in PO93XC− except for generation 7, and it decreased phenolic and flavonoid content in PO93XC− generation 3 (Figure 3a,c,e). The paclitaxel accumulating culture, P93AF+, accumulated significantly less paclitaxel on generation 7 (Table 1). For this generation, culture growth was surprisingly decreased from the unelicited control culture and flavonoid content remained unchanged (Figure 3b). Interestingly, the measured growth, flavonoid content and phenolic content in P93AF+ generation 1 did not change greatly because of methyl jasmonate accumulation despite seeing similar paclitaxel levels to generation 3 (Figure 3b,d,f). Although the long-term variability of paclitaxel accumulation has been published [13,23], this is the first study to simultaneously investigate culture growth and phenylpropanoid content across generations. These data illustrate the general instability of both conserved and specialized metabolism over time in both non-paclitaxel and paclitaxel accumulating cell lines.

However, some trend observations can be identified in these data. In general, methyl jasmonate elicitation affected the non-paclitaxel accumulating culture differently than the paclitaxel accumulating culture. In two of the three generations, PO93XC− exhibited less growth inhibition due to elicitation over the elicitation period compared to P93AF+. Additionally, methyl jasmonate increased phenolic and flavonoid content more in P93AF+ than PO93XC−, a trend that was observed in multiple cell lines in Figure 2. In fact, phenolic and flavonoid content in the PO93XC− culture were either unchanged or decreased in all but one time-point when compared to the unelicited culture. Overall, the paclitaxel accumulating cultures had a stronger response to methyl jasmonate than the non-paclitaxel accumulating lines. Snapshots of metabolic data over select generations, such as Figure 2 and Figure 3, show how metabolism varies over time and between non-paclitaxel and paclitaxel accumulating cultures.

### 2.3. The Relationship between Metabolite Accumulation and Aggregate Size

**Figure 4:**
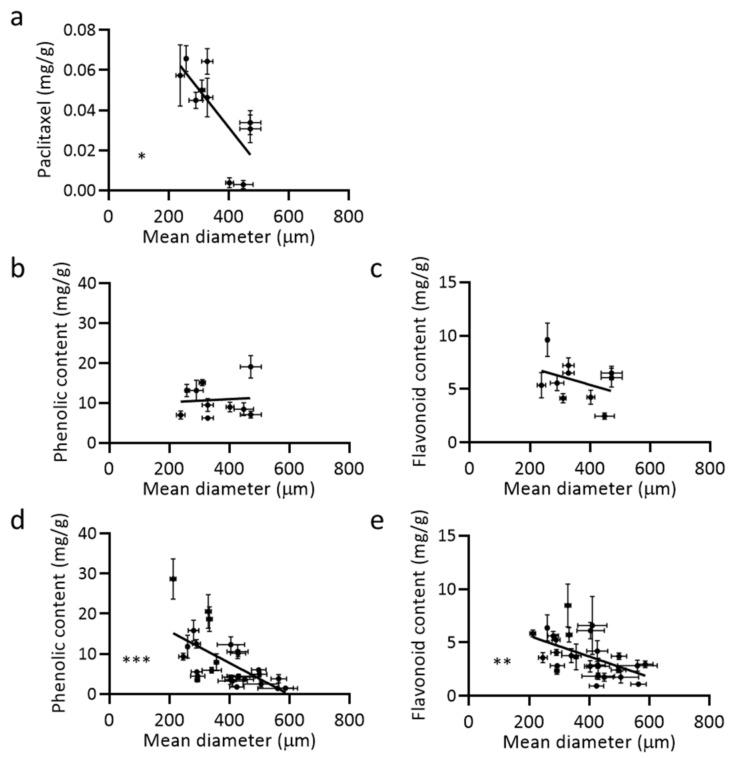
Correlation between mean aggregate size and metabolite accumulation is dependent on elicitation state. Paclitaxel (**a**), phenolic (**b**,**d**), and flavonoid (**c**,**e**) accumulation in elicited (**a**–**c**) and unelicited (**d**,**e**) P93AF+ cultures. Relationships were analyzed using the Pearson correlation coefficient (r); (a: *n* = 10, r = −0.742), (b: *n* = 10, r = 0.079), (c: *n* = 10, r = −0.364), (d: *n* = 27, r = −0.614), and (e: *n* = 27, r = −0.527). Each data point is the average of 3 biological replicates. (Error bars = standard deviation) (* *p* ≤ 0.05, ** *p* ≤ 0.01, *** *p* ≤ 0.001).

It is well established that paclitaxel accumulation is negatively correlated with aggregate size; smaller aggregates accumulate more paclitaxel [12,13]. In correlation with metabolite accumulation, the paclitaxel biosynthetic enzymes are more highly expressed in smaller aggregates [17]. This phenomenon was also observed in phenylpropanoid production through lignin staining in methyl jasmonate elicited non-paclitaxel and paclitaxel accumulating cultures (Figure 2e). Consolidation of data from the previous studies allowed assessment of average aggregate size and specialized metabolite accumulation. The relationship between mean diameter and metabolite content was analyzed using the Pearson correlation coefficient (r), a statistic to measure the linear correlation between −1 and +1. −1 is a strong inverse correlation, 0 is no correlation, and +1 is a strong positive correlation. Although there were no observable trends in the non-paclitaxel accumulating culture (PO93XC−) (data not shown), the paclitaxel accumulating culture (P93AF+) results were surprising. As expected, paclitaxel accumulation negatively correlated with average aggregate size in methyl jasmonate P93AF+ (r = −0.742) (Figure 4a). However, when elicited, phenolic and flavonoid accumulation in P93AF+ did not strongly correlate with aggregate size (r = 0.079 and r = −0.364, respectively) (Figure 4b,c). Additionally, interesting, unelicited P93AF+ cultures maintained a significant inverse correlation between basal phenolic and flavonoid content and aggregate size (r = −0.614 and r = −0.527, respectively) (Figure 4d,e).

Paclitaxel accumulating *Taxus* cultures have basal levels of phenolic and flavonoid compounds, which are elevated through methyl jasmonate elicitation, as seen in Figure 2 and Figure 3. It is not totally surprising that these basal levels are correlated to aggregate size. The increase in phenolic and flavonoid concentration in smaller aggregates could be due to a more favorable microenvironment: more nutrient availability, increased cell-to-cell signaling, and decreased susceptibility to surface shear forces [12]. However, it is unclear why this relationship would dissipate in an elicited state. Perhaps under methyl jasmonate elicitation, biosynthesis of more potent specialized metabolites, such as paclitaxel, takes precedence amongst cellular resources, which is tightly correlated to aggregate size under these conditions. Furthermore, the taxane and phenylpropanoid biosynthetic pathways could be separately co-regulated by factors outside of methyl jasmonate elicitation, which would explain their unique responses with respect to aggregate size. It was recently reported that shear forces can be used to decrease aggregate size in a predictable and controlled manner, providing opportunity to further engineer the *Taxus* production system [30]. Application of this technique with transcriptomic analysis could provide a method to further understand the effect of elicitation state and aggregate size especially in pathways other than taxane biosynthesis.

## 3. Materials and Methods

### 3.1. Maintenance and Treatment of Taxus Cultures

Three *Taxus cuspidata* cell lines (P93AF, PO93X, PO93XC) and one *Taxus canadensis* cell line (CO93D) were obtained from the United States Plant Soil and Nutrition Laboratory in Ithaca, NY. Cultures were maintained in media containing 20 g/L sucrose, 0.5 mg/L naphthaleneacetic acid, 22.5 µg/L benzylaminopurine, and 3.21 g/L Gamborg B5 Basal Medium with Vitamins (Caisson Laboratories, Smithfield, UT, USA) Media was autoclaved at 121 °C for 30 min and upon cooling, supplemented with a filter-sterilized antioxidant solution (5% *v*/*v*) containing 2.5 g/L ascorbic acid, 2.5 g/L citric acid, and 14.6 g/L L-glutamine. Suspension cultures were incubated in the dark, shaking at 125 rpm at 23 °C in an Innova S44i Biological Shaker (Eppendorf, Hauppauge, NY, USA).

P93AF+ and CO93D+ are paclitaxel accumulating lines and are denoted as so with + after their name in text. PO93X− and PO93XC− do not accumulate UPLC-detectible levels of paclitaxel in either an unelicited or elicited state and, therefore, are labeled with—after their name in text. All chemicals, unless otherwise specified, were purchased from Sigma Aldrich (St. Louis, MO, USA). Cells were maintained in an Erlenmeyer flasks approximately 2.5 × culture volume, and sub-cultured on a bi-weekly basis, as previously described [12].

Cultures were treated on day 7 of the culture period, and subsequent sampling occurred on the day designated in the text and figures. Samples were taken by pipetting 1 mL of well mixed culture containing cells and media into a microcentrifuge tube. Samples for metabolite (paclitaxel, phenolic, or flavonoid) analysis were stored at −80 °C, and samples for lignin staining were processed immediately. Cultures were elicited with 200 µM methyl jasmonate suspended in 50% ethanol and filtered through 0.2 µm PVDF membrane. Control groups were mock elicited with sterile 50% ethanol.

### 3.2. Long-Term Experimental Set-Up

Two cell lines, PO93XC− and P93AF+ were used in this experiment. On day 14 of the culture period, 20 mL of well-mixed cell culture were removed from each control flask and mixed in one sterile container. This combination of unelicited cultures was used to inoculate new flasks, then designated as the next generation of the cell culture. On day 14, the culture combination and inoculation process was repeated to establish the next generation, and so on for nine generations total. Half of the cultures per generation was elicited with 200 µM methyl jasmonate, and unelicited cultures were used to begin the next generation. Every other generation was sampled for paclitaxel quantification, but only generations 1, 3, and 7 were analyzed for culture growth and phenolic and flavonoid content.

### 3.3. Biomass Measurements Using the Coulter Counter

All biomass and aggregation data were measured using the Multisizer 3™ Coulter counter equipped a 2000 µm aperture (Beckman Coulter, Brea, CA, USA), as previously described [31]. In brief, 2 mL of well-mixed culture were diluted to 500 mL with an electrolyte solution containing 0.32 g/L NaN_3_, 6.34 g/L NaCl, and 65% glycerol for analysis. The reported number and size of aggregates were converted to biomass using the correlation for *Taxus* cultures [31].

### 3.4. Phenolic and Flavonoid Content Assays

1 mL well-mixed culture samples stored in the −80 °C freezer, were thawed and dried overnight in an evaporative centrifuge. Pellets were resuspended in 1 mL acidified methanol (0.01% acetic acid in methanol) using a combination of shaking, vortexing, sonicating in a sonication bath, and manually dissociating with a stainless-steel spatula. When the pellets were evenly and sufficiently pulverized to sand-like granules, the non-dissolved cell matter was separated from the methanol extract by centrifuging at 20,000× *g* for 10 min. This sample processing method was also used for paclitaxel quantification.

The phenolic content per sample was determined using the Folin–Ciocalteu reagent assay [32]. 20 μL methanol extract was combined with 40 μL Folin–Ciocalteu reagent (0.2 N) and 160 μL sodium carbonate (700 mM) in a microcentrifuge tube. After 10 min incubation, any precipitant was pelleted via centrifugation 20,000× *g* for 1 min. 200 μL supernatant was transferred to a 96-well plate, and the absorbance was read at 750 nm using the accuSkan GO UV/Vis Microplate Spectrophotometer (Thermo Fisher Scientific, Waltham, MA, USA). Absorbance values were converted to mg/mL using gallic acid as a phenolic compound standard.

The flavonoid content per sample was determined using an aluminum chloride-based assay [33]. 25 μL methanol extract and 50 μL water were combined with 75 μL NaNO_2_ (6 g/L) in a 96-well plate. After 1 min incubation, 75 μL AlCl_3_∙6H_2_O (22 g/L) was added to the wells. After 2 min incubation, 75 μL of NaOH (0.8 M) was added to each well, and the absorbance was read at 490 nm. Absorbance values were converted to mg/mL using catechin as a flavonoid compound standard.

### 3.5. Paclitaxel and Baccatin III Quantification

Paclitaxel and baccatin III accumulation was quantified using the Waters Acquity UPLC H-Class system (Waters, Milford, MA, USA) using a sample processing method previously described [34]. 7 and 14 days after treatment, 1 mL well-mixed culture samples were dried in an evaporative centrifuge. Organic compounds were isolated via methanol (acidified with 0.01% acetic acid) extraction and resuspended in 25% methanol, 35% acetonitrile, and 40% water. Compound separation was performed using a 1.7 µm Acquity UPLC BEH C18 column (Waters). A 10 µL sample injection was separated by a 4 min gradient from 30% to 80% acetonitrile with a flow rate of 0.35 mL/min. Data were processed and analyzed using the Empower 2 software (Waters). The area under the peak at 228 nm was converted to mg/L using commercially available standards of paclitaxel and baccatin III (Alfa Aeser, Haverhill, MA, USA).

### 3.6. Lignin Staining

Lignin staining was performed using an acidified phloroglucinol assay [35]. To prepare the assay solution, 2 g phloroglucinol were dissolved in 30 mL ethanol solution (20%), and then 20 mL HCl was added. 200 μL of sample was transferred to a slide as an unstained control. The remaining supernatant was removed from the rest of the cell and replaced with 500 μL phloroglucinol assay solution. After 5 min incubation, 200 μL of sample was transferred to a slide and imaged.

## 4. Conclusions

This work uniquely investigated metabolism in *Taxus*, as the first to use non-paclitaxel accumulating cultures further understand the interface between phenylpropanoid and taxane biosynthesis. In general, methyl jasmonate limited culture growth while increasing biosynthesis of taxanes and phenylpropanoids in paclitaxel accumulating cultures. However, the effects of methyl jasmonate elicitation were quite different in non-paclitaxel accumulating cultures, with elicitation typically leading to no change or even reduce accumulation of these compounds. Interestingly, aggregate size was correlated to paclitaxel levels, but not to phenylpropanoid levels in elicited cultures. Aggregate size was correlated to phenylpropanoid levels at basal conditions. These data suggest that specialized metabolism pathways, while regulated by similar signals, can be manipulated separately, which could lead to engineering tactics to enhance paclitaxel accumulation by limiting flux through competing pathways. This research was conducted at the metabolite level; supplementation with transcriptomic and proteomic data can be used to identify direct targets to complete this goal. This approach to optimize *Taxus* culture considers specialized metabolism beyond the product of interest, paclitaxel, and has the potential to significantly advance metabolic engineering efforts in *Taxus* and other important plant systems where specialized metabolic pathways compete for resources.

## Figures and Tables

**Table 1:** Paclitaxel accumulation (mg/g dry weight) in the cell line P93AF+ over nine generations. (PE = post-elicitation) (*n* = 3 biological replicates, ± = standard deviation).

Generation	7 Days PE (mg/g)	14 Days PE (mg/g)
1	0.05 ± 0.005	0.05 ± 0.004
3	0.03 ± 0.007	0.06 ± 0.006
5	0.03 ± 0.006	0.05 ± 0.009
7	0.002 ± 0.002	0.003 ± 0.002
9	0.07 ± 0.006	0.06 ± 0.02

## Data Availability

Not applicable.
